# Relationship between EGFR gene mutation and local metastasis of resectable lung adenocarcinoma

**DOI:** 10.1186/s12957-017-1103-x

**Published:** 2017-03-03

**Authors:** Yunqiang Nie, Wei Gao, Na Li, Wenjun Chen, Hui Wang, Cuiyun Li, Haiyan Zhang, Ping Han, Yingmei Zhang, Xin Lv, Xinyi Xu, Hongyan Liu

**Affiliations:** 1 Department of Respiratory Medicine, Linyi People’s Hospital, Linyi, 276000 China; 2 Department of Blood Screening Test, Linyi People’s Hospital, Linyi, 276000 China; 3 Department of Oncology, Linyi People’s Hospital, Linyi, 276000 China; 4 Department of Pathology, Linyi People’s Hospital, Linyi, 276000 China; 5 Department of Medicine, Linyi People’s Hospital, Linyi, 276000 China

**Keywords:** Lung adenocarcinoma, Endobronchial metastasis, Bronchial grade, Bronchoscopy, EGFR

## Abstract

**Background:**

Resectable lung adenocarcinoma is dominated by peripheral distribution, and surgical resection is the main treatment protocol. However, high recurrence rate remains after surgery. Lung adenocarcinoma with epidermal growth factor receptor (EGFR) mutation has strong invasion ability, but the effects of this mutation on local invasion in early lung adenocarcinoma have been rarely studied. This study aimed to assess the effects of EGFR mutation on local invasion in resectable lung adenocarcinoma.

**Methods:**

A retrospective analysis of 103 patients clinically diagnosed with peripheral lung adenocarcinoma was included. They underwent preoperative bronchoscopy, which indicated grades 2 or 3 bronchial involvement (lumen of the lobe or segment). The associations of EGFR mutation with pleural invasion, endobronchial metastasis, and lymph node metastasis were analyzed according to pathologies of pleural invasion and lymph node metastasis, as well as EGFR gene mutation detected by postoperative pathological specimens. Statistical analyses were performed by unpaired Chi-square test using the SPSS16.0 software.

**Results:**

In patients with EGFR mutation, pleural invasion, endobronchial metastasis, and lymph node metastasis rates were 62.5, 39.1, and 34.4%, respectively, indicating statistically significant differences (*p* = 0.003). Meanwhile, the pleural invasion rate in patients with wild-type EGFR was 43.6%, significantly reduced compared with patients with mutated EGFR (62.5%; *p* = 0.018). In addition, the endobronchial metastasis rate in patients with wild-type EGFR was 17.9%, significantly lower than in patients with EGFR mutation (39.1%; *p* = 0.005). However, lymph node metastasis rates were similar between EGFR mutated and wild-type patients (34.4 vs 25.6%, respectively, *p* > 0.05).

**Conclusions:**

Early resectable lung adenocarcinoma patients with EGFR mutation showed a higher rate of local invasion compared with those harboring wild-type EGFR. This finding provides a basis for improved therapy.

**Trial registration:**

This study was supported by Project of Medical and Health Science Technology in Shandong Province (2015WS0376)

## Background

Computed tomography (CT) diagnosis rate for early peripheral lung adenocarcinoma is increasingly higher, and surgical resection of lung segment and lobe is more popular. However, high recurrence rate remains after surgery. Selection of the surgical margin is one of the key factors affecting recurrence. The EGFR gene affects the invasion capability of lung adenocarcinoma. However, how EGFR mutation helps early lung adenocarcinoma invade local structures that has been rarely studied, and it is not known whether it can provide a basis for treatment in early lung adenocarcinoma. In this study, bronchial nvasion was accurately assessed by preoperative bronchoscopy, combined with postoperative pathologies of pleural and lymph node metastases as well as EGFR gene mutation assays, to further elucidate local invasion in early resectable lung adenocarcinoma. Our data provide a strong basis for improved therapy.

## Methods

### Patients

A retrospective analysis of 103 outpatients or inpatients treated at Linyi People’s Hospital, between January 2011 and August 2015, were selected. They presented with sputum, cough, chest pain, fever or blood sputum, and were pathologically diagnosed with lung adenocarcinoma postoperatively. They included 50 men (59.38 ± 10.038 years old), and 53 women (57.73 ± 11.706 years old). Among them, there were 30 smokers, including 25 men and 5 women. The patients underwent high-resolution CT (HRCT) examinations, and lesions were found in the peripheral lung. Patients were tolerable to bronchoscopy and surgery.

### Examination methods

1. All patients underwent 64-sliced CT (Light Speed VCT 64, GE Company Fairfield USA) plain and enhanced scans, with layer thickness and interval of 0.625 mm. The CT scanning ranged from the superior aperture of thorax to the costophrenic angle. Plain scans were first performed, followed by bolus injection of contrast agent with a high-pressure syringe (iopromide 300, 300 mgI/ml; flow rate, 3 ml/s; total amount, 80 ml; cubital vein injection). Enhanced scans were conducted in delayed stage (30 s after venous stage). Three-dimensional reconstruction was performed if necessary; the horizontal plan was considered the routine layer. Peripheral or central lung cancer was initially determined. All patients underwent bronchoscopic examination (BF260, Olympus, Tokyo, Japan) and grades 2 or 3 (lumen of the lobe or segment) bronchial invasions were found. The surgical specimens were routinely fixed with neutral formalin, paraffin-embedded, and H&E stained for routine pathological examination. Postoperative lung cancer was classified according to the seventh edition of the TNM classification of lung cancer in 2009 [1]. All preoperative patients were confirmed no metastasis by PET-CT, or cerebral MRI, bone ECT, and abdomen ultrasound.
2. EGFR gene detection:
  Postoperative pathological sections were used to assess 18, 19, 20, and 21 exon mutation on ARMS-PCR (scorpion amplification refractory mutation system) [2] with Gene mutation detection kit purchased from Amoy Diagnostics Co. Ltd. (Xiamen, China).

### Evaluation criteria

According to the pathological results, pleural invasion was defined to extend beyond the elastic pleural layer, and lymph node metastasis was diagnosed by postoperative lymph node pathology. Endobronchial metastasis referred to lumen of the lobe or segment (2 or 3 grade bronchus) invaded by peripheral lung adenocarcinoma, or other bronchial lumens were invaded beyond the lobe containing the tumor. Features of metastasis bronchus: mucosal hyperemia and edema, infiltration change, polypoid change, etc.; meanwhile, lung adenocarcinoma was confirmed by endoscopic biopsy and pathology. All patients underwent a bronchoscopic examination, performed by expert pulmonologists using an Olympus EVIS bronchovideoscope BF260 (Olympus, Tokyo, Japan). If no significant endobronchial lesions were found in the most distal bronchi by bronchoscopy, the patient was considered to have no endobronchial metastasis. However, if a tumor mass or infiltration was visible by bronchoscopy, the patient was considered to have endobronchial metastasis. Bronchial grading was defined as follows: tracheal, 0; right and left main bronchus, 1; lobar bronchus, 2; segmental bronchus, 3.

### Surgical procedure

In the VATS procedure, all patients underwent general anesthesia with double lumen endotracheal intubation and single lung ventilation. The patient was placed in the lateral decubitus position. Thoracoscopic lobectomy was performed through three or four incisions. We placed a thoracoscope in the 7th or 8th intercostal space in the middle or anterior axillary line. The main operating port of incision was located at the anterior axillary line in the 4th or 5th intercostal space, with a length of about 3 cm; the port just facing the lung hilus was the best. A 1.5 cm auxiliary operating port was placed in the auscultation triangle. The lung and pleural space were carefully inspected for unexpected metastatic disease. According to the various fissure situations, the management sequence of pulmonary arteries, veins, and bronchia was different. Pulmonary artery, vein, bronchus, and fissure were divided using an Endoscopic Linear Cutter. The pulmonary artery with smaller diameter (<7 cm) was ligated using Hem-o-lok. Lymph node dissection was performed by standard procedure, removing all accessible Hilar and mediastinal lymph nodes, including levels L2–L4, and L7–L11 on the right side, and levels L4–L11on the left side, according to the American Thoracic Society classification.

For thoracotomy, a posterolateral skin incision ranging in length from 20 to 25 cm was made. The latissimus dorsi and serratus anterior muscles were dissected along the fifth intercostal space. Thoracotomy was performed at the fifth intercostal space, with the fifth rib sectioned at the costovertebral angle. A standard rib retractor was inserted. All resections were performed in a conventional manner. Hilar and mediastinal lymph node dissection was performed in all patients, and the extent of mediastinal lymph node dissection was the same as in the VATS group. One chest tube was placed at the posterior aspect of the chest cavity.

### Statistical analysis

Statistical analyses were performed by unpaired Chi-square test using the SPSS16.0 software. *p* < 0.05 was considered statistically significant.

## Results

According to postoperative pathological TNM staging, there were 52, 24, and 27 stages I, II, and IIIA cases, respectively. Patients with EGFR mutation represented 62.14% (64/103); 37.86% (39/103) harbored wild-type exon.

### Local invasion

A total of 60 cases with pleural invasion were found, including 43 and 17 patients with mutated and wild-type EGFR, respectively; there were 32 cases of lymph node metastasis, including 22 and 10 patients with mutated and wild-type EGFR, respectively. A total of 32 cases of endobronchial metastasis were found, including 25 with mutated EGFR and 7 with wild-type gene.

**Fig. 1:**
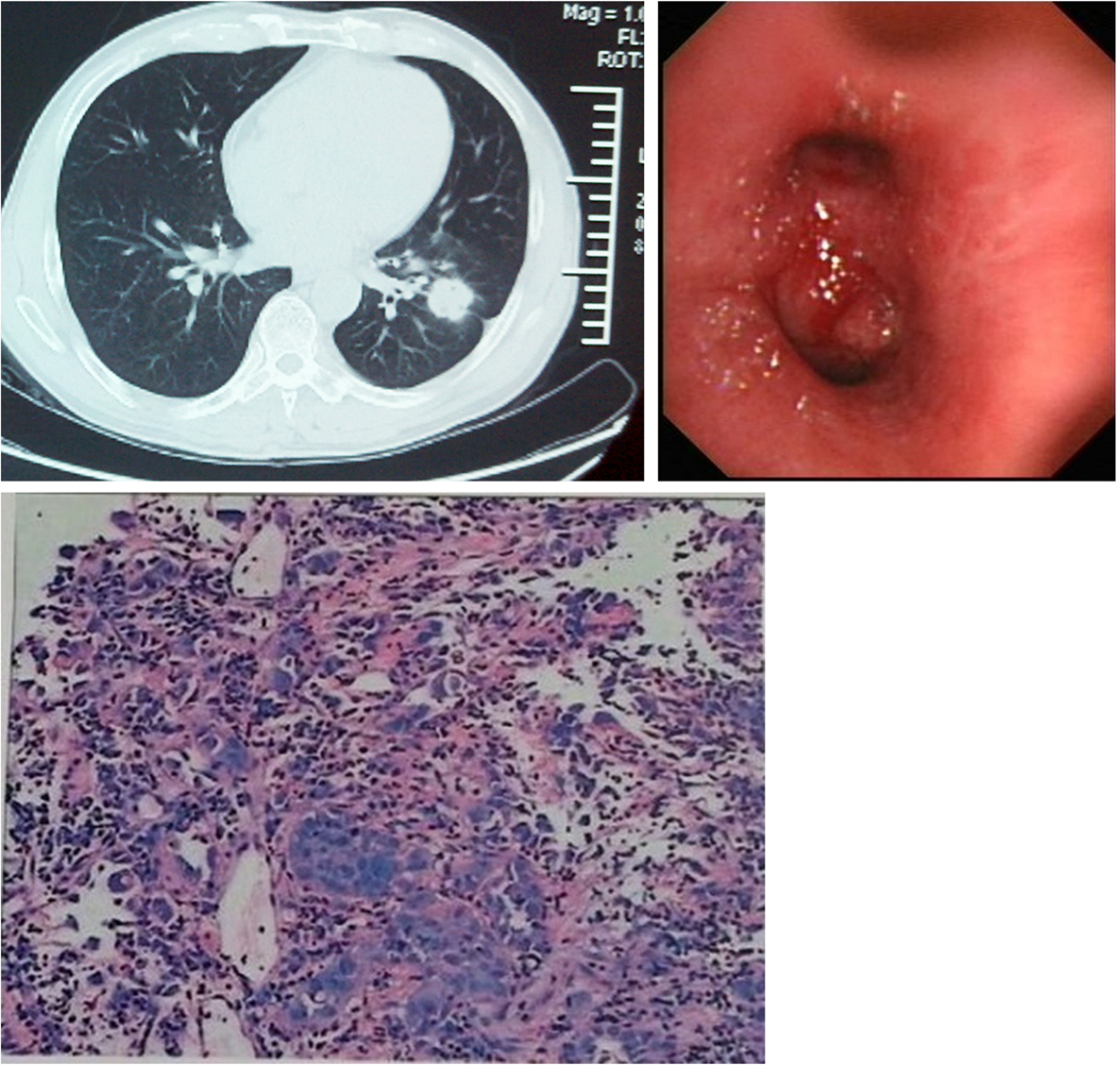
CT features and endobronchial metastasis. Endobronchial metastasis in peripheral lung adenocarcinoma. CT scan showing lung cancer in the medial anterior basal segment of left lower lobe, with pleural retraction. Appearance obtained by bronchoscopy. Left lung lower lobe and upper lobe bronchial mucosal hyperemia, infiltration change, and luminal stenosis. Endoscopic biopsy pathology, adenocarcinoma; EGFR mutation, exon 19 deletion mutation

Among the 64 patients with EGFR mutation, pleural invasion, endobronchial metastasis, and lymph node metastasis rates were 62.5% (43/64), 39.1% (25/64), and 34.4% (22/64), respectively, showing statistically significant differences (*p* = 0.003). For the 39 wild-type patients, pleural invasion, endobronchial metastasis, and lymph node metastasis rates were 43.6% (17/39), 17.9% (7/39), and 25.6% (10/39), respectively, indicating statistically significant differences among the three groups (*p* = 0.038). Interestingly, pleural invasion (*p* = 0.018) and endobronchial metastasis (*p* = 0.005) rates in patients with EGFR mutation were significantly higher compared with those obtained for individuals harboring wild-type EGFR. However, the difference in lymph node metastasis rates between both groups was not statistically significant (*p* > 0.05) (Tables 1 and 2) (Fig. 1).

**Table 1:** Relationship between local invasion and gene mutation status

Metastasis	Mutated *N* = 64			Wild-type *N* = 39	*P* value (mutated vs. wild-type)
	Exon 18 *N* = 2	exon 19 *N* = 36	exon 21 *N* = 26		
Pleural invasion	1	22	20	17	*p* = 0.018
Lymph node metastasis	0	12	10	10	*p* > 0.05
Endobronchial metastasis	1	16	8	7	*p* = 0.005

**Table 2:** Amounts of grades 2 or 3 bronchus invasion

Diameter	Total	Endobronchial metastasis (grades 2 or 3 bronchus)	No endobronchial metastasis (grades 2 or 3 bronchus)
<2 cm	31	5	26
2.1–3 cm	28	7	21
3.1–5 cm	26	10	16
5.1–7 cm	10	7	3
>7.1 cm	8	3	5

### Relationship between EGFR gene mutation and pathological staging

p-stage I incidence was highest (54.7%, 35/64) in patients with EGFR mutations, followed by p-stage IIIA (28.1%,18/64), and p-stage II (17.2%, 11/64), indicating statistically significant differences among the three stages (*p* < 0.001). For patients without EGFR mutation, p-stage I, p-stage II, and p-stage IIIA incidence rates were 43.6, 33.3, and 23.1%, respectively, and the differences were not statistically significant (*p* = 0.158) (Table 3).

**Table 3:** Effects of EGFR mutation on pathological staging

	p-stage I	p-stage II	p-stage IIIA
Mutated	35	11	18
Wild-type	17	13	9

### Surgical methods

Three patients underwent sleeved lobectomy, including 2 and 1 with mutated and wild-type EGFR, respectively; 8 patients had pulmonary wedge resection, including 4 in each group; 3 patients underwent segmentectomy, including 1 and 2 with mutated and wild-type EGFR, respectively; 85 patients underwent lobectomy, including 31 harboring wild-type EGFR and 54 with mutated gene; 4 patients underwent two lobe combination resection, including 3 and 1 with mutated and wild-type EGFR. 4 patients (3 patients with pulmonary wedge resection and 1 patient with two lobe combination resections) had positive margin resection, including 1 with wild-type gene and 3 harboring mutated EGFR (Table 4).

**Table 4:** Relationship between the surgical method and EGFR

EGFR	Lobectomy	Segmentectomy	Wedge resection	Sleeved lobectomy	Two lobe combination resection
Mutate-type	54	1	4	2	3
Wild-type	31	2	4	1	1

## Discussion

With recent advances in diagnostic techniques for lung cancer, detection rate is increasingly higher, and more and more resectable lung adenocarcinomas are found in early stage. In this study, postoperative patients with p-stage I and EGFR mutation accounted for 54.7% (*p* = 0.000), followed by p-stage IIIA (28.1%, 18/64), and p-stage II (17.2%, 11/64). In addition, wedge resection, pulmonary segmentectomy and lobectomy were increasingly used, while invasion of peripheral structure by early lung adenocarcinoma affects postoperative recurrence or treatment [3].

Common local metastases of peripheral lung adenocarcinoma include pleural, endobronchial and lymphatic metastases. Chest CT can be used to assess lymph node enlargement and tumor diameter, but is not sensitive enough to differentiate bronchial mucous membrane and visceral pleural invasion. Postoperative specimens are used to determine pleural and lymph node invasions, but cannot help identify endobronchial metastasis. Since early lung adenocarcinoma can expand along the bronchus [4], bronchoscopy can accurately assess endobronchial metastasis; however, the traditional endoscopic examination aims mainly to obtain pathological diagnosis and does not guide in selecting a surgical procedure for peripheral lung adenocarcinoma in the aspect of endobronchial metastasis [5]. In this study, endobronchial metastasis was defined as grades 2 or 3 invasions in central bronchial anatomic regions in patients with lung adenocarcinoma. Since lobectomy is more difficult in cases with tumor invading lobar bronchus opening or adjacent principal bronchus [6], the sleeved protocol is superior in increasing the length of bronchial incision [7]. When metastatic tumors are observable by bronchoscopy, there may be a high possibility of positive stump after simple lobectomy [8–10]. The recurrence rate after wedge resection is higher than that after pulmonary segmentectomy, while the local recurrence after segmentectomy is more common than when lobectomy is performed [11–15]. Endobronchial metastasis is significantly increased in patients with EGFR mutation, in agreement with our data presented above 39.1 vs 17.9% in patients with mutation and wild-type EGFR, respectively; (*p* = 0.005). Patients mutated for EGFR might present higher recurrence and decreased 5-year survival rates [16], with better efficacy to chemoradiotherapy after surgery than other types of metastasis [17].

Pleural invasion rate was highest in peripheral lung adenocarcinoma, with patients harboring EGFR mutation showing significantly higher values (62.5%) compared with those without EGFR mutation (*p* = 0.018). Moreover, recurrence rate after wedge resection was shown to be high in lung adenocarcinoma patients with stage I and pleural invasion [15, 18]. This study confirmed a stronger local invasion in patients with EGFR gene mutation, with significantly higher pleural and endobronchial metastases compared with patients without mutation. Furthermore, pleural and endobronchial metastases were significantly more common than lymph node metastasis. Invasion of local structure in early lung adenocarcinoma was to a certain extent associated with postoperative recurrence, which can help provide a basis for surgical treatment. Since p-stage I lung adenocarcinoma incidence is significantly increased in patients with EGFR mutation, it is critical to select an appropriate surgical procedure, especially that with significant effects on the incision margin and recurrence.

Sole TNM staging is unable to predict early lung adenocarcinoma invasion, unlikely to identify recurrence in patients after surgical resection [19, 20], and unable to predict chemotherapeutic efficacy. The relationship between EGFR mutation and peripheral structure invasion can provide a basis for comprehensive therapy. Indeed, postoperative patients with EGFR mutation show higher efficacy rates to targeted therapy and chemotherapy compared with wild-type patients [21]. Patients harboring EGFR mutation are prone to recurrence in the incision margin [22–24], and have a high metastasis rate [25]; however, they still show higher survival after recurrence [26–28]. For instance, the 5-year survival rate in patients with EGFR mutation was shown to be higher than that of cases without mutation [21], which may be related to drug sensitivity and comprehensive treatment. Therefore, clarifying the clinical characteristics of early lung adenocarcinoma patients with EGFR mutation aims to provide a basis for clinical treatment.

The limitation of this study lies in that only patients with operable lung adenocarcinoma were assessed, not including other types of lung cancer. Therefore, further multi-factor, large sample, and multi-center studies are needed to confirm the association of local structure invasion in early lung adenocarcinoma with EGFR.

## Conclusions

Compared with early resectable lung adenocarcinoma patients harboring wild-type EGFR, those with EGFR mutation showed a higher rate of local invasion, with pleural invasion most common. In addition, endobronchial metastasis incidence was higher than that of lymph node metastasis, suggesting the influence of EGFR mutation on resection scope.

## Abbreviations

EGFR: Epidermal growth factor receptor
